# Fluorescence lifetimes and choriocapillaris flow deficits in intermediate age-related macular degeneration: a cross-sectional study

**DOI:** 10.1186/s12886-026-05159-z

**Published:** 2026-07-25

**Authors:** M. Tarhan, N. L. Jungk, T. Brockmann, D. Meller, M. Hammer

**Affiliations:** 1 Department of Ophthalmology, University Hospital Jena, Am Klinikum 1, 07747 Jena, Germany; 2 Clinical Optometry, Department of Sciences and Technology (SciTec), Ernst Abbe University of Applied Sciences Jena, Jena, Germany; 3 Department of Ophthalmology, University Medicine Rostock, Rostock, Germany; 4 Center of Medical Optics and Photonics, Friedrich Schiller University Jena, Jena, Germany

**Keywords:** AMD, Fluorescence lifetime imaging, OCT angiography, Choriocapillaris, Flow deficit, Retinal metabolism

## Abstract

**Purpose:**

To assess the association between retinal fluorescence lifetimes (τm) and choriocapillaris (CC) flow deficits (FD) in intermediate age-related macular degeneration (AMD).

**Methods:**

Twenty-six pseudophakic eyes with intermediate, non-exudative AMD (mean age 83 ± 5 years) underwent fluorescence lifetime imaging ophthalmoscopy (FLIO) and optical coherence tomography angiography (OCTA). Fluorescence lifetimes were recorded in short- and long-wavelength channels and analyzed across ETDRS subfields. CC flow deficits were quantified using local thresholding. Associations between τm and FD were evaluated using Spearman’s rank correlation.

**Results:**

Mean τm values were consistently higher in the long-wavelength channel compared to the short-wavelength channel across all subfields. Mean CC FD increased from the outer ring (0.50 ± 0.07) to the central subfield (0.65 ± 0.11). Correlation coefficients between τm and FD ranged from − 0.07 to 0.37, with no statistically significant associations (all *p* > 0.05).

**Conclusions:**

No statistically significant association was observed between fluorescence lifetimes and choriocapillaris flow deficits in this exploratory cohort. Although both biomarkers are known to be altered in AMD, the absence of evidence for a statistically significant spatial association suggests that FLIO and OCTA may reflect complementary aspects of disease pathology. Larger studies incorporating structural retinal biomarkers and longitudinal follow-up are warranted.

**Clinical trial registration:**

Not applicable.

## Introduction

Age-related macular degeneration (AMD) is a leading cause of irreversible central vision loss in elderly populations [27]. The disease involves complex metabolic and microvascular alterations affecting the retinal pigment epithelium (RPE), Bruch’s membrane, and the choriocapillaris [7, 12]. Despite extensive knowledge of these pathogenic pathways, their interrelationship in vivo during intermediate AMD remains incompletely understood.

In early and intermediate AMD, structural and metabolic alterations precede visual loss. Lipid-rich drusen accumulate between the RPE and Bruch’s membrane, disrupting nutrient exchange and triggering local inflammation [7, 12, 23]. Thickening of Bruch’s membrane reduces diffusion of oxygen and nutrients, contributing to relative hypoxia in the outer retina [1, 14, 19].

The choriocapillaris (CC) supplies oxygen and nutrients to the RPE and photoreceptors, which have among the highest oxygen consumption rates in the body [14, 28]. Histologic and imaging studies demonstrate early CC alterations in AMD, often adjacent to drusen [1, 4, 17]. OCT angiography (OCTA) enables in vivo visualization of the CC, and increasing flow deficits have been reported with disease severity [2, 24]. However, whether CC alterations precede or follow RPE degeneration remains debated [1, 16].

At the level of the RPE, chronic oxidative and metabolic stress leads to alterations in endogenous autofluorescent compounds. Changes in fluorophore composition and microenvironment are thought to reflect altered cellular metabolism and oxidative processes relevant to AMD progression [12, 13].

Fluorescence lifetime imaging ophthalmoscopy (FLIO) measures fluorescence decay times (τ), reflecting the biochemical microenvironment of retinal fluorophores [10, 21, 22]. Unlike intensity-based autofluorescence imaging, FLIO provides information on molecular composition and environment that is less dependent on fluorophore concentration [10, 20]. Prolonged fluorescence lifetimes have been reported in AMD, suggesting alterations in retinal metabolism and oxidative stress [9, 18].

Both prolonged fluorescence lifetimes and increased choriocapillaris flow deficits have been described in AMD. However, it remains unclear whether these biomarkers reflect spatially coupled disease processes or complementary aspects of AMD pathology. Clarifying this relationship may improve the interpretation of multimodal imaging in intermediate AMD. If both biomarkers reflect closely related pathophysiological mechanisms, a spatial association would be expected within corresponding retinal regions. Therefore, the aim of this exploratory pilot study was to investigate whether retinal fluorescence lifetimes measured by FLIO are associated with OCTA-derived choriocapillaris flow deficits within corresponding ETDRS subfields. We hypothesized that, if metabolic alterations and microvascular impairment are spatially linked, corresponding FLIO and OCTA parameters would demonstrate measurable associations.

## Methods

This prospective cross-sectional pilot study was conducted in accordance with the Declaration of Helsinki. Written informed consent was obtained from all participants. Inclusion criteria were pseudophakic eyes with intermediate AMD (AREDS classification). Exclusion criteria included neovascular AMD, geographic atrophy, pachychoroid-associated diseases (including pachychoroid pigment epitheliopathy), other retinal pathology, and insufficient image quality. One eye per subject was included.

FLIO measurements were performed using a Spectralis-based system (Heidelberg Engineering, Heidelberg, Germany) with a 473 nm picosecond laser diode (80 MHz). Fluorescence photons were detected via time-correlated single-photon counting (SPC-150; Becker & Hickl GmbH, Berlin, Germany) in a short-wavelength channel (SSC, 498–560 nm) and a long-wavelength channel (LSC, 560–720 nm). FLIO provided 30° field images at a resolution of 256 × 256 pixels. Photon arrival histograms were fitted with a series of three exponential decay functions using SPCImage software, and amplitude-weighted mean lifetimes (τm) were calculated. Regional values were averaged within ETDRS subfields to enable standardized comparison.

**Fig. 1:**
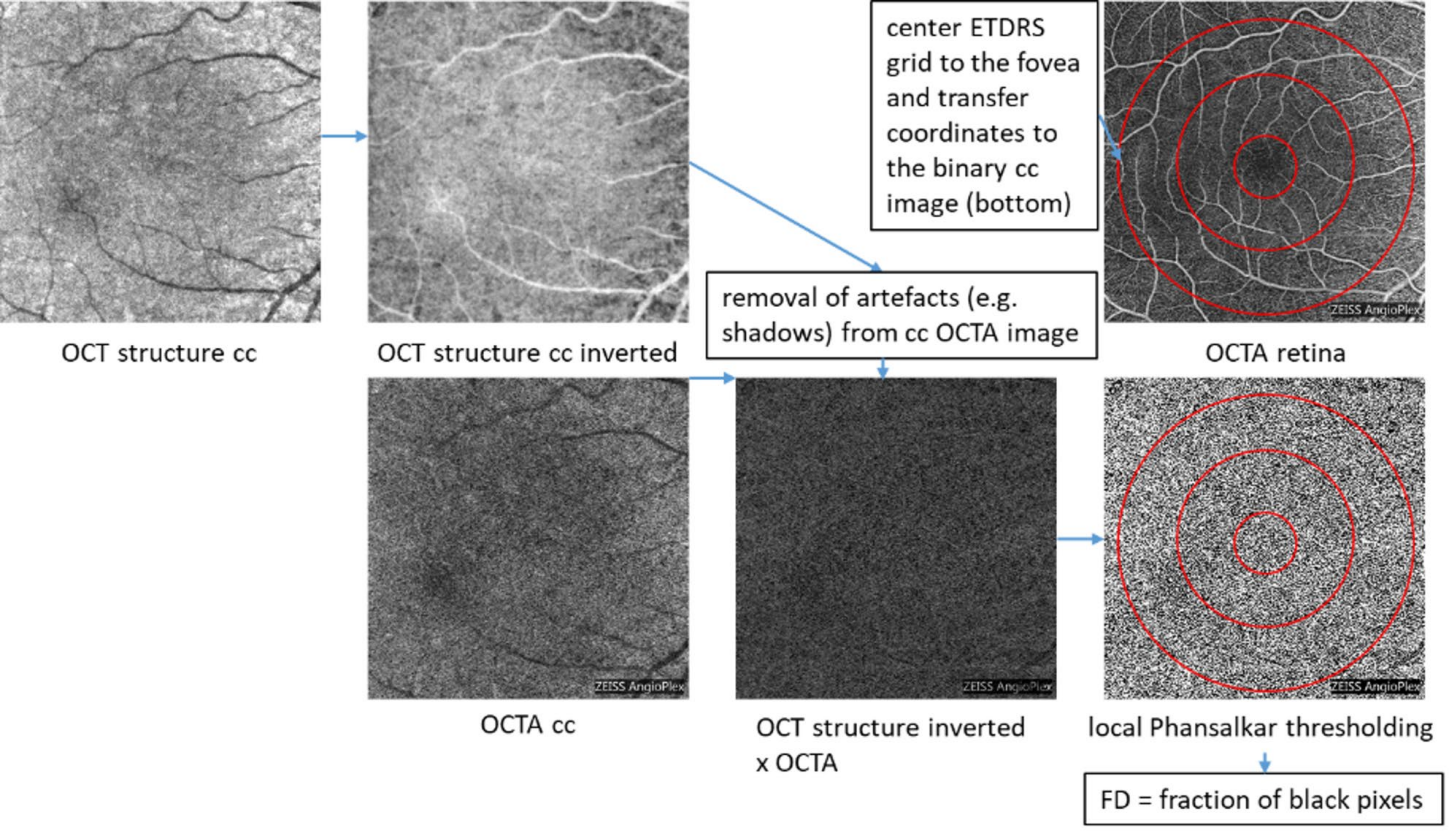
CC OCTA workflow. Representative compensated CC slab (6 × 6 mm) segmented 21–41 μm beneath the RPE. The OCTA flow image was multiplied by the inverse structural OCT image to reduce shadowing, followed by Phansalkar local thresholding. FD was quantified within ETDRS subfields (central, inner ring, outer ring)

OCTA (6 × 6 mm) was performed using a Cirrus 5000 HD-OCT system (Carl Zeiss Meditec, Germany). Image scaling was automatically corrected for ocular magnification by the device software using axial length correction. The CC slab (21–41 μm below the RPE; thickness 20 μm) was automatically segmented using the device software. To reduce attenuation-related artifacts, OCTA images were processed using established approaches for choriocapillaris visualization and quantitative analysis [3, 5, 26]. Flow deficits were calculated after Phansalkar local thresholding [6] and expressed as the proportion of non-perfused pixels within ETDRS subfields. The analysis workflow is illustrated in Fig. 1.

Statistical analyses were performed using IBM SPSS Statistics 26 (IBM Corp., Armonk, NY, USA). Continuous variables are presented as mean ± standard deviation (SD). Associations between fluorescence lifetimes (τm) and choriocapillaris flow deficits (FD) were evaluated using Spearman’s rank correlation coefficient (r), given the non-parametric distribution of the data.

Correlations were analyzed across corresponding ETDRS subfields (central, inner ring, and outer ring). A two-tailed p-value < 0.05 was considered statistically significant. Given the exploratory design, no formal adjustment for multiple comparisons was performed.

## Results

Twenty-six pseudophakic eyes (15 female, 11 male; mean age 83 ± 5 years) with intermediate, non-exudative AMD were included. All eyes fulfilled AREDS criteria and provided FLIO and OCTA images of sufficient quality for quantitative analysis.

**Fig. 2:**
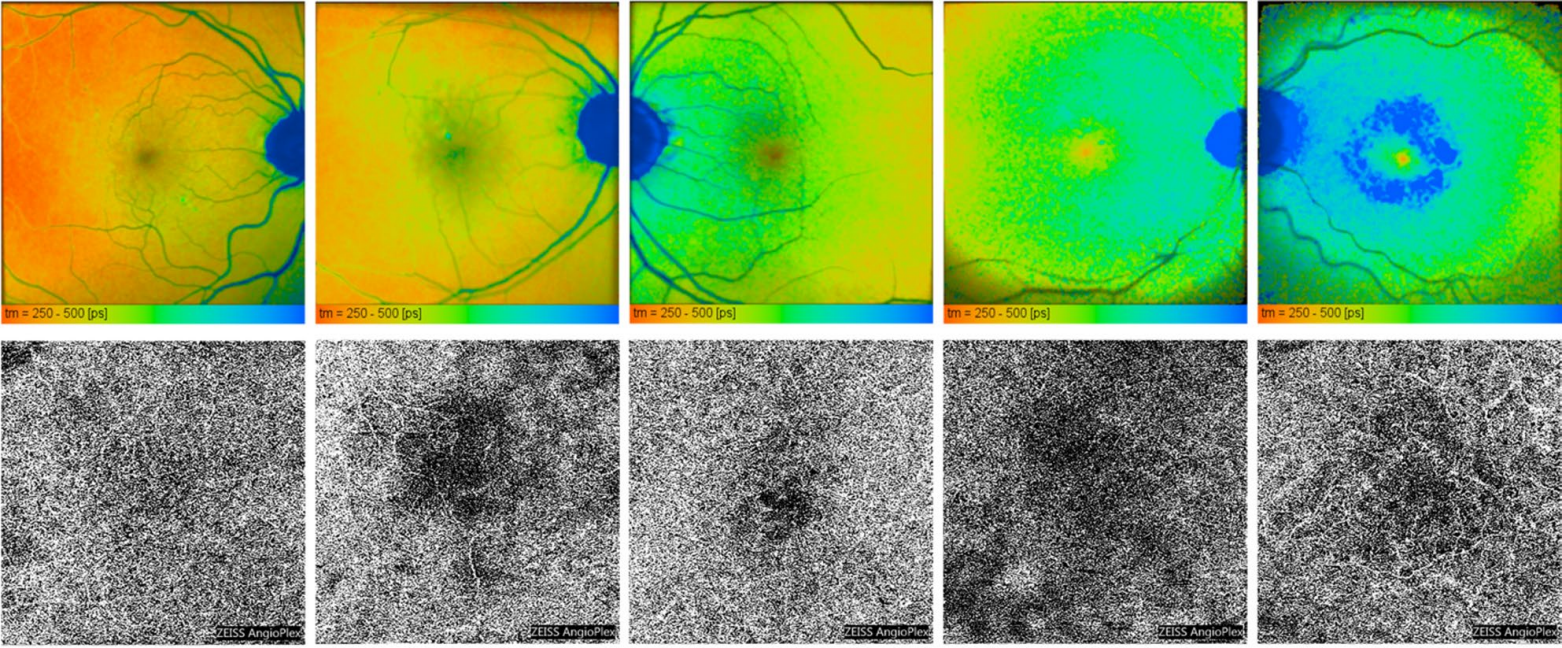
Intensity-weighted mean fluorescence lifetime (τm) maps of the macular region acquired in the long spectral channel (LSC, 560–720 nm) from representative eyes with intermediate AMD (top). Warmer colors indicate shorter lifetimes, whereas cooler colors indicate longer lifetimes. Corresponding macular AngioPlex OCT angiography images of the same eyes are shown below (bottom)

FLIO imaging demonstrated consistently longer fluorescence lifetimes in the long-spectral channel (LSC) compared to the short-spectral channel (SSC) across all ETDRS subfields (Table 1). In the LSC, mean fluorescence lifetimes increased from 322 ± 56 ps in the central subfield to 368 ± 65 ps in the outer ring. In contrast, SSC lifetimes were shortest centrally (213 ± 51 ps) and highest in the inner ring (252 ± 40 ps). Spatial fluorescence lifetime maps revealed heterogeneous distributions with focal areas of prolonged lifetimes (Fig. 2, top), corresponding to localized retinal alterations. No extreme lifetime values exceeding 1000 ps were observed.

**Table 1:** Mean values and standard deviations of τm and CC FD across ETDRS subfields (*n* = 26)

Parameter	Mean	Standard Deviation
CC FD Central	0.65	0.11
CC FD Inner Ring	0.59	0.09
CC FD Outer Ring	0.50	0.07
SSC-τm Central (ps)	212.81	50.56
SSC-τm Inner Ring (ps)	252.42	39.62
SSC-τm Outer Ring (ps)	241.77	24.09
LSC-τm Central (ps)	322.42	56.28
LSC-τm Inner Ring (ps)	359.62	49.03
LSC-τm Outer Ring (ps)	368.19	64.87

OCTA processing followed a standardized workflow for choriocapillaris analysis, including compensation for signal attenuation and local thresholding (Fig. 1). En face CC slabs displayed dense capillary networks interspersed with scattered flow voids. Quantitative analysis showed a gradual increase in flow deficit (FD) from the outer ETDRS ring (0.50 ± 0.07) towards the foveal center (0.65 ± 0.11), with the highest values observed centrally (Table 1). No consistent quadrant-specific or hemispheric asymmetries in CC perfusion were identified.

Across all ETDRS subfields, Spearman correlation analysis revealed no statistically significant associations between fluorescence lifetimes (SSC or LSC) and CC FD (all *p* > 0.05) (Table 2). Correlation coefficients ranged from weakly negative to moderately positive values (*r* = − 0.07 to 0.37) without a consistent directional pattern. Several associations approached statistical significance, particularly between central and inner subfield parameters (e.g., SSC central vs. FD inner ring: *r* = 0.37, *p* = 0.06), but did not reach the predefined threshold.

**Table 2:** Spearman correlation coefficients (r) τm and CC FD across ETDRS subfields (*n* = 26)

Parameter	CC FD Central	CC FD Inner Ring	CC FD Outer Ring
SSC-τm Central	0.33 (*p* = 0.10)	0.37 (*p* = 0.06)	0.37 (*p* = 0.06)
SSC-τm Inner Ring	0.14 (*p* = 0.49)	0.31 (*p* = 0.13)	0.28 (*p* = 0.17)
SSC-τm Outer Ring	−0.07 (*p* = 0.74)	0.05 (*p* = 0.81)	0.02 (*p* = 0.93)
LSC-τm Central	0.26 (*p* = 0.20)	0.33 (*p* = 0.10)	0.37 (*p* = 0.07)
LSC-τm Inner Ring	0.10 (*p* = 0.62)	0.25 (*p* = 0.21)	0.26 (*p* = 0.21)
LSC-τm Outer Ring	0.14 (*p* = 0.51)	0.09 (*p* = 0.66)	0.07 (*p* = 0.74)

## Discussion

In this exploratory cross-sectional study, no statistically significant associations were identified between FLIO-derived fluorescence lifetimes and OCTA-derived choriocapillaris flow deficits across ETDRS subfields in eyes with intermediate AMD. The study was designed to investigate whether two imaging biomarkers known to be altered in AMD show spatial correspondence within affected eyes. Within the regional averaging approach applied here, no evidence for a direct spatial association was identified.

FLIO-derived fluorescence lifetimes provide information on the biochemical microenvironment of endogenous retinal fluorophores, but they do not represent a single retinal layer or a single fluorophore. Instead, amplitude-weighted mean lifetimes reflect a composite signal influenced by multiple endogenous fluorophores and retinal structures [10, 21, 22]. Previous studies have demonstrated associations between FLIO-derived fluorescence lifetimes and AMD-related structural alterations, particularly drusen [9]. The present study specifically focused on the relationship between FLIO-derived fluorescence lifetimes and OCTA-derived choriocapillaris perfusion rather than on structural OCT biomarkers.

Prolonged lifetimes have been associated with oxidative stress, changes in fluorophore composition, and altered cellular metabolism [10, 21]. In contrast, OCTA-derived flow deficits reflect perfusion changes within the choriocapillaris and are considered a marker of microvascular integrity [2, 24]. Given these fundamentally different biological substrates, a direct spatial correlation between both parameters cannot be assumed.

Current models of AMD pathogenesis emphasize the interplay of multiple processes, including RPE dysfunction, extracellular deposit formation, inflammation, and choriocapillaris impairment [11, 25]. These processes may not evolve synchronously. It is therefore plausible that metabolic alterations detectable by FLIO either precede or follow microvascular changes captured by OCTA, thereby limiting the ability to detect associations in a cross-sectional setting.

Methodological aspects may further contribute to the absence of detectable correlations. Intermediate AMD is characterized by pronounced spatial heterogeneity, with focal lesions such as drusen occupying limited retinal areas. ETDRS-based regional averaging provides a standardized framework for multimodal comparison but may reduce sensitivity to detect localized structure–function relationships. As a result, potential associations confined to lesion sites may be attenuated when analyzed at the regional level.

Intrinsic regional differences across ETDRS subfields may influence absolute biomarker values, as reflected by the higher flow deficit values observed centrally in the present cohort. Consequently, correlations based on absolute subfield values may partly reflect regional baseline variation rather than disease-specific coupling between FLIO and OCTA parameters. Nevertheless, the use of ETDRS-based regional averaging may have reduced sensitivity to detect focal lesion-based associations between FLIO and OCTA parameters.

In addition, both FLIO lifetimes and OCTA-derived flow deficits are subject to biological variability and measurement noise. The exploratory character of this pilot study, together with the relatively small sample size of 26 eyes, may have limited the statistical power to detect weak associations. While no significant correlations were identified, weak or region-specific relationships cannot be excluded and may become evident in larger or longitudinal datasets.

Another limitation is the absence of an age-matched healthy control group. Previous studies have independently demonstrated altered fluorescence lifetimes and choriocapillaris perfusion abnormalities in AMD compared with healthy eyes. However, the primary objective of the present study was not to compare AMD with healthy eyes, but to investigate whether two AMD-associated imaging biomarkers show spatial correspondence within eyes with intermediate AMD. In healthy eyes, OCTA-derived flow deficit measurements predominantly reflect physiological regional and interindividual variability rather than AMD-related microvascular impairment. Therefore, a correlation between fluorescence lifetimes and disease-related flow deficits would not necessarily be expected in a healthy control group. Nevertheless, this was not examined in the present study, and future studies including healthy controls may help contextualize regional biomarker variation.

Technical limitations of OCTA should also be considered. Structural alterations such as drusen-related RPE elevations may influence the delineation of the choriocapillaris slab and thereby affect flow deficit quantification [3, 26]. Although compensation strategies were applied to reduce signal attenuation, residual inaccuracies related to segmentation and projection artifacts may persist.

Finally, differences in depth sensitivity between both modalities may contribute to the observed findings. FLIO integrates fluorescence signals across multiple retinal layers, predominantly from the RPE and outer retina, whereas OCTA selectively visualizes a thin slab corresponding to the choriocapillaris. This anatomical mismatch further complicates direct comparisons between metabolic and vascular parameters.

Structural OCT biomarkers such as drusen volume, pigment epithelial detachment characteristics, outer retinal thickness, or RPE abnormalities were not incorporated into the present analyses. Such parameters may influence both fluorescence lifetimes and choriocapillaris flow deficit quantification and could further improve the biological interpretation of multimodal imaging. Their evaluation was beyond the scope of the present exploratory study and should be addressed in future investigations.

The lack of a direct association between fluorescence lifetimes and choriocapillaris perfusion is consistent with previous observations of dissociation between structural, functional, and vascular biomarkers in retinal and neurodegenerative diseases [8, 15, 29]. The retina, as an accessible extension of the central nervous system, may provide a useful model for studying such neurovascular interactions.

In conclusion, this exploratory pilot study did not identify statistically significant associations between FLIO-derived fluorescence lifetimes and OCTA-derived choriocapillaris flow deficits in intermediate AMD. Although both biomarkers are independently altered in AMD, the absence of evidence for a statistically significant spatial association suggests that they may reflect complementary rather than directly coupled aspects of disease pathology. Larger longitudinal studies integrating structural OCT biomarkers and lesion-based analyses are warranted to further clarify the temporal and spatial relationship between metabolic and microvascular alterations during AMD progression.

## Data Availability

The datasets generated and/or analyzed during the current study are not publicly available due to patient privacy and institutional restrictions but are available from the corresponding author on reasonable request.
